# Use of a composite, 3D‐printed patch as a partial airway replacement: A pilot study on the porcine model

**DOI:** 10.1002/btm2.70103

**Published:** 2026-01-27

**Authors:** Marco Mammana, Alessandro Gandin, Giovanni Zambello, Margherita Pelosin, Alberto Elmi, Domenico Ventrella, Silvia Todros, Veronica Torresan, Federica Pezzuto, Marco Pietra, Noemi Romagnoli, Andrea Dell'Amore, Maria Laura Bacci, Fiorella Calabrese, Giovanna Brusatin, Federico Rea

**Affiliations:** ^1^ Thoracic Surgery Unit, Department of Cardiac, Thoracic, Vascular Sciences and Public Health University of Padua Padua Italy; ^2^ Department of Industrial Engineering University of Padua Padua Italy; ^3^ INSTM Padova RU University of Padua Padua Italy; ^4^ Department of Veterinary Medical Sciences Alma Mater Studiorum – University of Bologna Bologna Italy; ^5^ Pathology Unit, Department of Cardiac, Thoracic, Vascular Sciences and Public Health Padua University Hospital Padua Italy

**Keywords:** 3D printing, biomaterials, tracheal replacement

## Abstract

Tracheal replacement is an unmet clinical need, as patients with long or complex airway defects are managed with tracheostomy or permanent stents. Experimental and clinical research is ongoing in order to find safe airway substitutes; however, the strategies under investigation suffer from major limitations, such as unsatisfactory re‐epithelialization, insufficient long‐term mechanical support, and complex ex vivo procedures. A ready‐to‐use and stable patch, able to support airway functionality and tissue regeneration, remains a significant challenge. Here we present the development of an off‐the‐shelf composite patch consisting of a resorbable polymer to aid epithelial restoration and a 3D‐printed multimaterial structure to guarantee effective mechanical stability. To evaluate the prosthesis performance, we designed a pilot study on a large animal setting, monitoring postoperative survival and airway healing for up to 60 days. An anterior cervical tracheal defect was created on four domestic pigs and patched with the prosthesis. The results were satisfactory in terms of postoperative survival, as only one animal died before the end of the study. However, endoscopic findings revealed a worsening stenosis due to wound contraction, granulation tissue formation, and partial displacement of the prosthesis. These findings were confirmed at histology, where a prominent inflammatory infiltrate was evident. Blood tests performed during follow‐up did not reveal any systemic inflammatory reaction. Overall, we believe that further optimization of the prosthesis design and materials is necessary in order to create an ideal “off‐the‐shelf” tracheal substitute. Nevertheless, this pilot study provides promising results and novel insights into a clinically relevant research area.

**Translational Impact Statement**

Tracheal replacement is a significant unmet need in modern medicine. Many alternatives have been proposed, often relying on decellularization and ex vivo re‐epithelization, but these have yielded inconsistent results. In this pilot study, we present a completely synthetic and multilayer tracheal prosthesis designed to fulfill the mechanical requirements for partial anterior defect repair and promote in situ epithelization. This off‐the‐shelf approach could be valuable for standardizing the synthesis of the prosthesis, as well as in emergency and life‐threatening situations where a ready‐to‐use option is required.

## 1 INTRODUCTION

Several pathological conditions such as tracheal stenosis, malacia, and primary or secondary airway neoplasms may require the resection of a tracheal segment and subsequent airway reconstruction. Typically, reconstruction involves reapproximating and anastomosing the healthy tracheal stumps. However, this procedure is only feasible as long as the airway segment to be removed is less than 5–6 cm long in adult patients.^1^ For longer defects, reapproximation of the tracheal stumps is not possible due to the excessive tension on the airway anastomosis and the consequent risk of dehiscence. Currently, the only established therapeutic option in such cases is permanent stenting of the airway, which causes significant morbidity and an impairment of quality of life.^2^

Over the years, investigators have experimented with various options for airway replacement, including synthetic materials, allografts, tracheal transplantation, autologous tissue composites, and bioengineering.^3^ Some of these tracheal replacement strategies have shown promising results in humans^4^, ^5^; however, none of them are yet established treatment options.

Tracheal allotransplantation requires long and complex procedures involving weeks of heterotopic revascularization to establish a functional blood supply, as well as the extended use of immunosuppressants after the graft.^6^ Moreover, lack of adequate mechanical support often requires the use of a stent.^3^

Recently, tissue engineering has emerged as a valuable approach, with decellularized extracellular matrix gaining attention as a natural scaffold that preserves the native composition.^7^ Although it has been proven to be biocompatible, the use of decellularized ECM presents several limitations. First, the decellularization process is a time‐consuming, multistep procedure, and achieving optimization in terms of yield and reproducibility is often elusive.^8^ Second, the harsh chemical, physical, or biological treatments required to effectively produce acellular scaffold may induce a weakening of the ECM structure leading to impaired mechanical properties,^9^ which can eventually result in collapse of the structure, insufficient ventilation, and suffocation.^10^, ^11^ Furthermore, cellular materials remaining from deficient decellularization can trigger immune responses.^12^

All the reported approaches share major limitations: the need for a donor, short‐term stability, and the inability to be used in an emergency setting.

Synthetic materials are therefore essential for the development of prostheses that can overcome these limitations.

We previously demonstrated the feasibility of repairing a posterior tracheal defect by interposing a sheet of synthetic, resorbable material and by buttressing the prosthesis with a pedicled muscle flap, on a consecutive series of eight patients affected by tracheoesophageal fistula, wherein conventional treatment with resection and end‐to‐end reconstruction was not feasible.^13^

However, this approach can only be used for posterior tracheal defects because the mechanical properties of available prostheses are insufficient to replicate those of the tracheal cartilage rings.

Based on this observation, we hypothesized that a composite prosthesis made from the same resorbable material that we have successfully used in clinical practice and coupled with a semi‐rigid inner layer could possess the optimal characteristics to be used as an airway substitute also for the cartilaginous trachea. Such a composite prosthesis would overcome many of the shortcomings associated with other tracheal replacement strategies, including time and availability constraints and would have the added benefit of being available off‐the‐shelf.

Therefore, we designed a pilot study to test the feasibility of repairing a limited, window‐like anterior trachea defect using such composite prosthesis on a large animal model, by observing postoperative survival and airway healing. We believe that the findings of this study may be used to inform on the feasibility of this approach for repair of longer and/or circumferential airway defects.

## 2 RESULTS

### 2.1 Composite prosthesis fabrication

**FIGURE 1:**
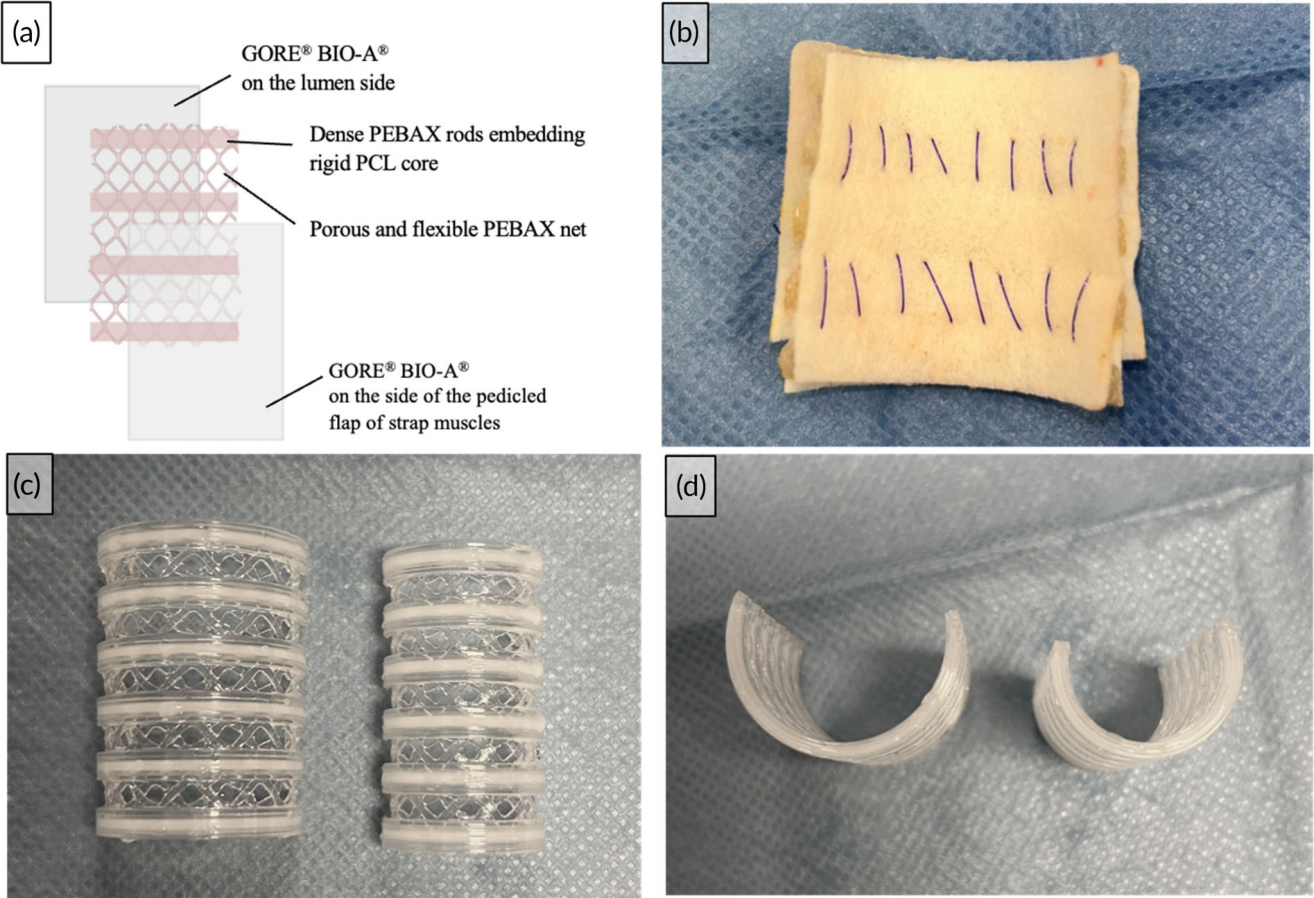
Design and fabrication of the multimaterial inner structure. The design of the composite scaffold (a) consists of three layers coupled together through adsorbable sutures. (b) The final constructs before implantation. Outer layers of Gore Bio‐A are sutured with purple PDS sutures to the inner, non‐degradable, supporting structure. The inner structure (b) is composed of dense sections and open meshes resembling the stiff cartilaginous rings and flexible annular rings, respectively. The inner structure replicates the curvature of native tracheal tissue (d).

We designed and fabricated a composite multimaterial prosthesis composed of three layers as reported in Figure 1a,b.

For the outer layers, we used a commercially available material (Gore Bio‐A tissue reinforcement; W.L. Gore & Associates, Inc., Newark, DE). This product is designed for reinforcement of soft tissue. The biodegradable polymer elicits a physiologic response, which fills the deficit with native tissue and gradually absorbs the device.^13^ When used to repair airway defects, this prosthesis showed optimal biocompatibility and did not result in airway stenosis or granulation tissue formation. Two months after surgery, respiratory epithelium was histologically demonstrated at the repair site.^14^

**FIGURE 2:**
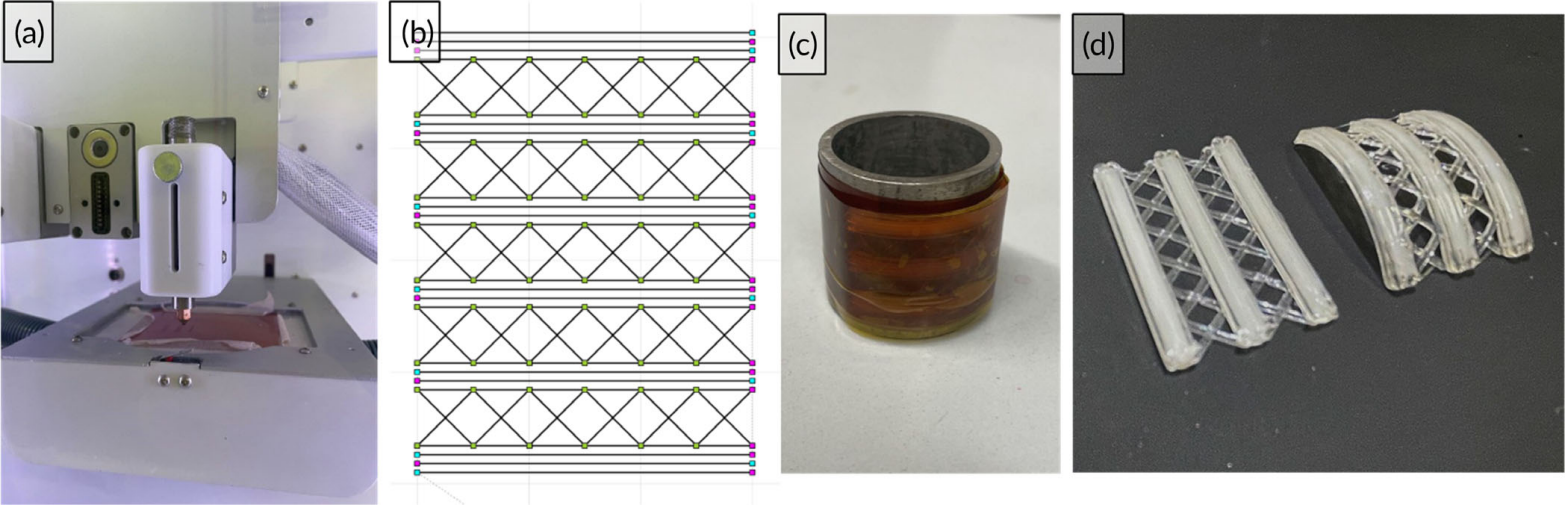
Fabrication of the inner non‐degradable structure composed of elastomeric PEBAX and rigid PCL. (a) Fabrication was performed as a flat structure exploiting AXO A3 printer equipped with two temperature‐controlled printing heads. 3D printer trajectories designed using SEL Program Generation Software are represented in (b). Printed structure is heated and curved on a stainless steel cylinder to mimic the natural shape of the trachea (c). (d) Representative images of composite structures before and after the shaping process.

The inner structure (Figure 1c,d), designed to mechanically support the prosthesis, has been optimized to provide the rigidity of the native cartilaginous rings alongside the flexibility of the annular ligaments. This structure was fabricated using multimaterial fused deposition modeling (FDM) 3D printing and consists of dense and rigid rings that are alternated with thin, open meshes (see Figure 2a,b). For the fabrication, we chose a commercial thermoplastic medical grade elastomeric copolymer, PEBAX 2533 MED. This technical copolymer, developed for biomedical applications such as urinary catheters, combines ease of fabrication and elastomeric properties. We adopted the medical grade version of the polymer which, according to the manufacturer, is designated as USP Class VI to minimize any potential immune response in the animal. Moreover, this designation, that is the most stringent with respect to polymers for medical devices, assured us the material to be extensively tested both in vitro and in vivo. To the best of our knowledge, this polymer, which imparts flexibility and elasticity to the structure, has not been used before for a tracheal prosthesis. Furthermore, to provide the proper mechanical stability, we opted to couple PEBAX with polycaprolactone (Purasorb PC 17) in sections resembling the cartilaginous rings. These rigid sections are composed of multilayer segments with an outer layer of PEBAX confining an inner PC 17 rod. The open mesh sections were fabricated with a single PEBAX layer and are designed to allow a large contact area between the Gore Bio‐A layers while also enabling the structure to deform. Finally, the structure is heated and curved around a cylindrical mold to properly adapt to the natural curvature of the trachea (see Figure 2c,d).

### 2.2 Mechanical characterization

**FIGURE 3:**
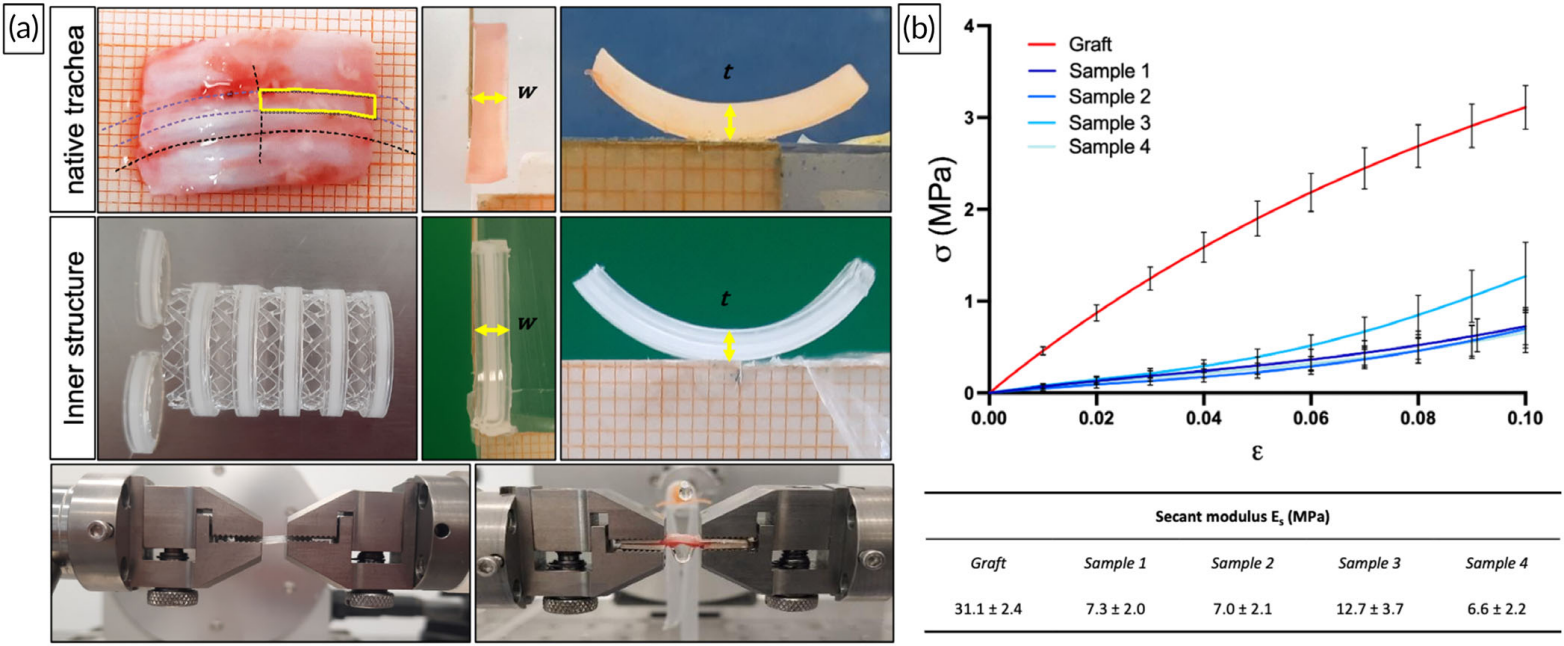
(a) Images of a tracheal tissue and inner structure specimens used to calculate relative width (w≈6mm) and thickness (t≈2mm). Specimens of the 3D‐printed inner structure for tensile tests were extracted from a single sample by cutting individual rigid segments of PCL and PEBAX along the circumferential direction. Samples are positioned between the grips of Bose ElectroForce® during the execution of the tensile test. (b) Results of the tensile tests performed on the native tracheal tissue and on the inner structure. Graph representing the average stress–strain curves for the 3D‐printed structure and native tracheal tissue samples (samples 1–4), with relative standard deviations. Average secant modulus (*E*_*s*_) and relative standard deviation reported are calculated at 10% strain.

Tensile tests were performed on porcine native cartilage tissue and the rigid, dense rings of the inner 3D‐printed structure to mechanically characterize them. As these regions function as the structure's mechanical support, they were chosen to define the load‐bearing properties of the native tissue and synthetic replacement. Specifically, three samples of native porcine trachea were tested from four different animals, while three samples were obtained from the curved prosthesis. Figure 3a shows examples of tracheal and 3D‐printed structure specimens. Mechanically, the prosthesis has been designed to provide a solid and prolonged support capable of preventing lumen collapse in the weeks following surgery. We therefore designed the cartilage‐mimicking sections of the prosthesis to exhibit moderately higher mechanical properties compared to the native tissue. Indeed, although a mechanical mismatch between the native tissue and synthetic implant could lead to stress concentration, irritation and local inflammation, collapsing is acknowledged as the primary failure mode of tracheal implants.^15^ This often results either in the need for an additional metallic stent, characterized by an elastic modulus far exceeding that of the native tissue, or in unavoidable lumen occlusion.^2^, ^9^, ^10^, ^11^ Moreover, a slightly elevated elastic modulus may help mitigate potential degradation mechanisms, such as oxidative damage or fatigue, which could otherwise compromise the structural support of the fabricated prosthesis. For this reason, we prioritized long‐term mechanical stability over achieving a perfect match between the mechanical properties of the native tissue and the prosthesis. Tensile tests validated the correct implementation of our design intent: elastic moduli obtained from native tissue samples range from 7 to 12 MPa (Figure 3b) consistently with previously reported data.^16^ Conversely, cartilage‐like sections of the 3D‐printed construct exhibited an elastic modulus on the same order of magnitude as the tracheal tissue but approximately three times higher (31 ± 2.4 MPa).

### 2.3 Postoperative outcomes

The surgical procedure was concluded without complication in all cases, and the animals recovered well from general anesthesia. In the following days, none of the animals showed signs of respiratory distress. However, one of them (animal n.3) was found dead on the morning of the 51st day after surgery. At autopsy, foamy hematic fluid was found inside the trachea, suggesting acute airway obstruction as the cause of death. All other animals survived until the predetermined endpoint of 60 days.

### 2.4 Surveillance bronchoscopy examination and blood tests

**FIGURE 4:**
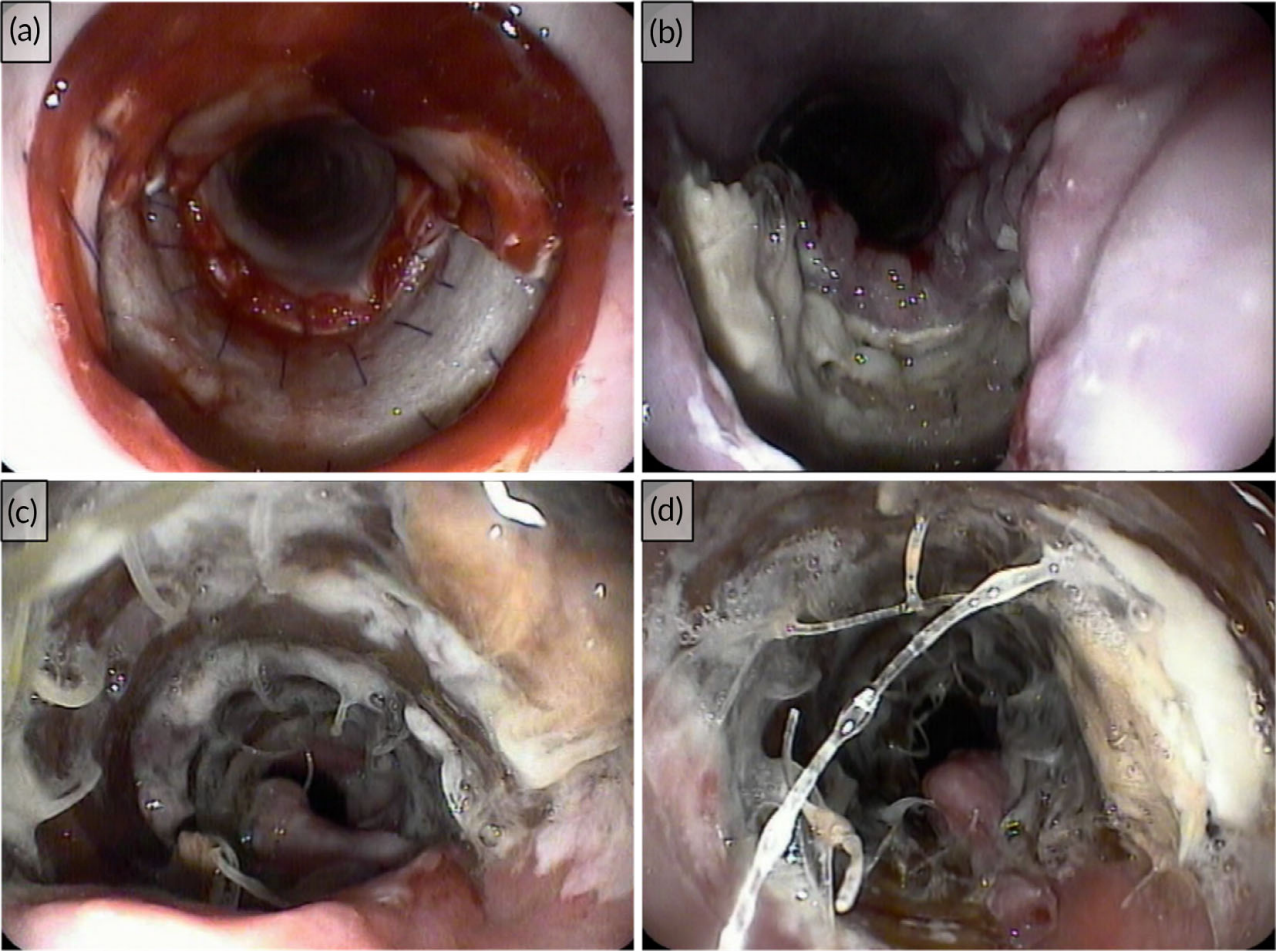
Bronchoscopy examination of the implanted after the surgical procedure (a) and 15 days (b), 30 days (c), and 60 days (d) after. Proper positioning can be seen right after the prosthesis application. Temporal examination of the implant shows early resorption of the innermost layer and poor integration of the non‐degradable layer with granulation tissue formation, shrinking of the tracheal lumen and consequent dislodgment of the prosthesis [rotated of approximately 180° in (c) and (d)].

**FIGURE 5:**
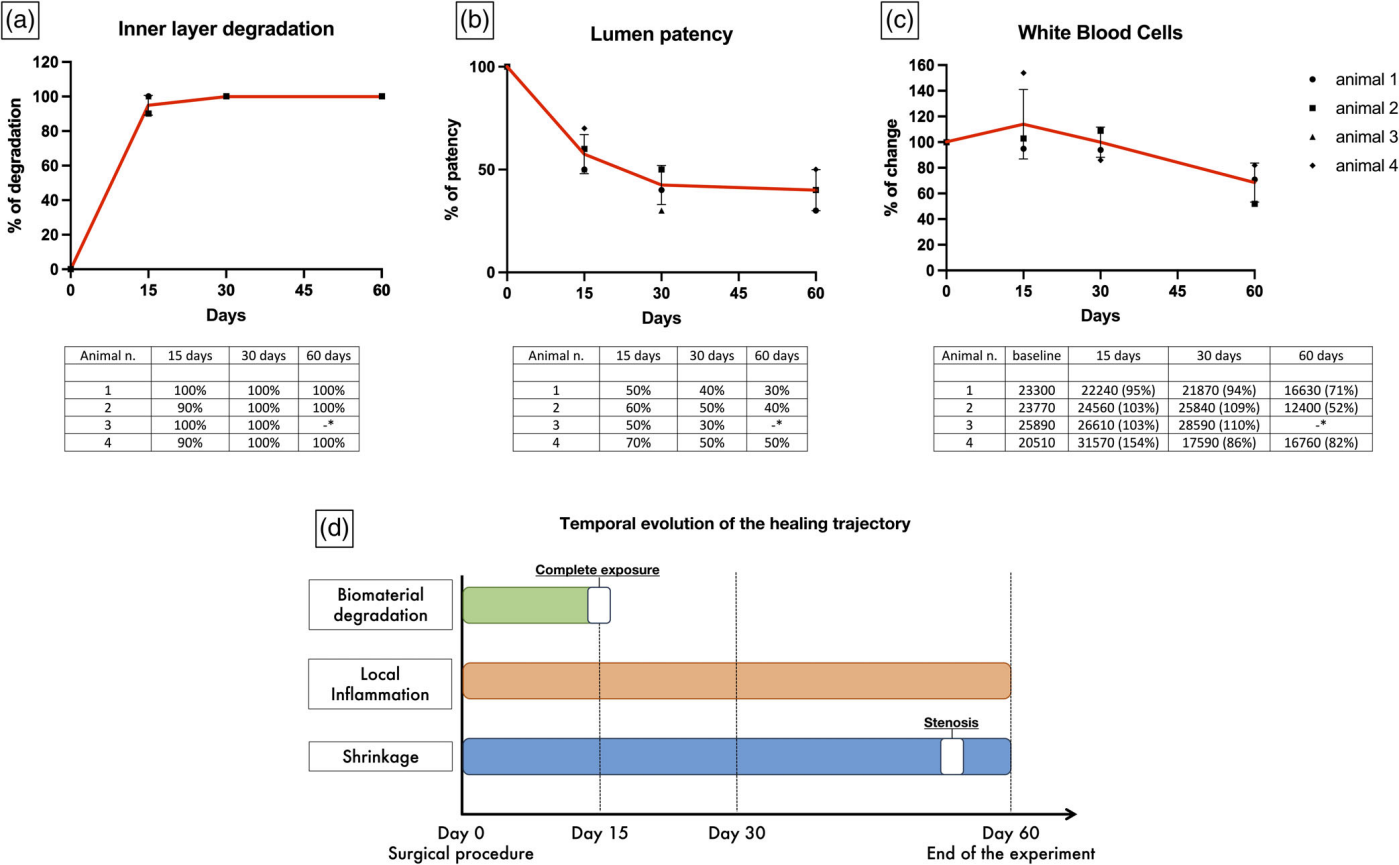
Evaluation of the inner layer degradation (a), airway stenosis (b), and white blood cells count (b) after surgery. Inner layer degradation is expressed as the percentage of degraded materials compared to the initial dimension. The degree of stenosis during follow‐up bronchoscopies is expressed as percentage of remaining lumen at the level of the window defect/lumen of the normal trachea. White blood cells are expressed as absolute value of cells/mm^3^ blood and (%) of baseline value. (*) Missing value due to death 51 days after surgery. Red lines reported in graphs connect the mean values in the analyzed timepoints calculated from values obtained from the four treated animals. Bars represent the data standard deviations. (d) Schematic representation of the temporal evolution of the mechanisms involved in the prosthesis failure after the surgical procedure.

Bronchoscopies performed at the end of the surgical procedure revealed that the prosthesis had maintained its curvature, with a normal airway lumen (Figure 4a). By the 15th postoperative day, the external layer had already almost completely resorbed, exposing the non‐resorbable mesh. The borders of the window defect progressively reapproximated, and, consequently, the prosthesis twisted and was displaced within the tracheal lumen. Granulation tissue formation at the edges of the defect was visible in most cases. A variable degree of stenosis was observed, caused by both the shrinking of the window defect, the prosthesis's bulk inside of the airway lumen, and the granulation tissue formation (Figure 4b–d). Airway stenosis tended to worsen over time. The mean (±SD) extent of external layer degradation, as visually assessed by the endoscopist, was 95% (±5.8%) at 15 days, and 100% (±0%) at subsequent (30 and 60 days) bronchoscopies (Figure 5a) while the mean (±SD) extent of lumen shrinkage was 57.5% (±9.6%) at 15 days, 42.5% (±9.6%) at 30 days, and 40% (±10%) at 60 days (Figure 5b). The mean (±SD) percent variation of the white blood cells count, compared to the baseline value, was 113.8% (± 27.1%) at 15 days, 99.8% (±11.7%) at 30 days, and 68.3% (±15.2%) at 60 days (Figure 5c). Similarly, IL‐6 levels were below the assay's detection limit at all time points (not shown). Overall, these results were compatible with no signs of systemic alterations or inflammation.

### 2.5 Gross and histologic examination of tracheal segments

**FIGURE 6:**
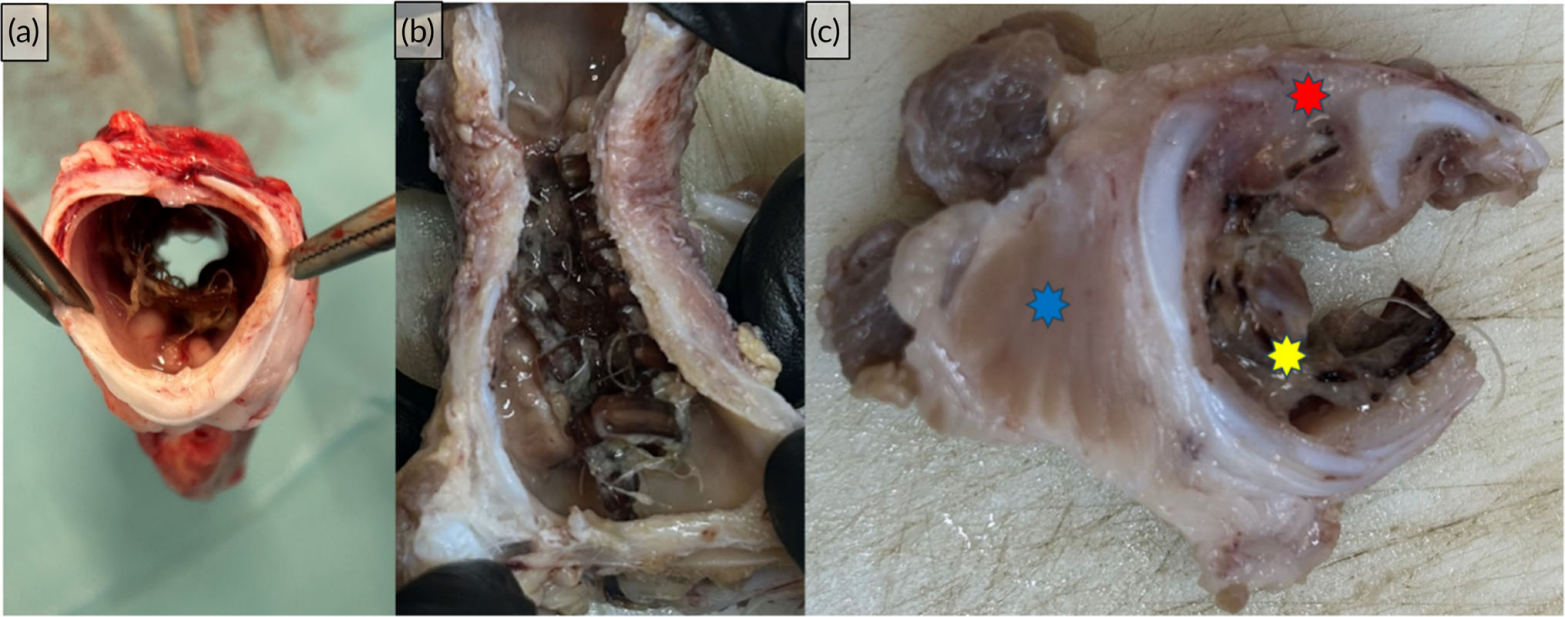
Macroscopic examination of the tracheal segment 2 months after application of the prosthesis. (a) Axial view of the specimen: a “triangle shape” deformity of the trachea is evident, due to shrinkage of the window defect created on its anterior part. (b) The specimen is incised longitudinally on its posterior side, allowing to appreciate the prosthesis from its luminal side. A lack of integration of the prosthesis with surrounding tissues is observed. (c) Transverse section of the trachea. From the inner to the outermost layer, it is possible to observe the prosthesis (yellow asterisk), the remaining cartilage defect (red asterisk), and the overlying muscle layer (blue asterisk). These three layers are now misaligned and the window defect created during surgery, originally occupying half of the tracheal circumference, has considerably shrunk.

Macroscopic examination of the tracheal segments demonstrated marked airway deformity due to shrinking at the defect site (Figure 6a). The prostheses showed poor integration with the host tissue, detaching easily from the luminal surface of the trachea as shown in Figure 6b,c.

**FIGURE 7:**
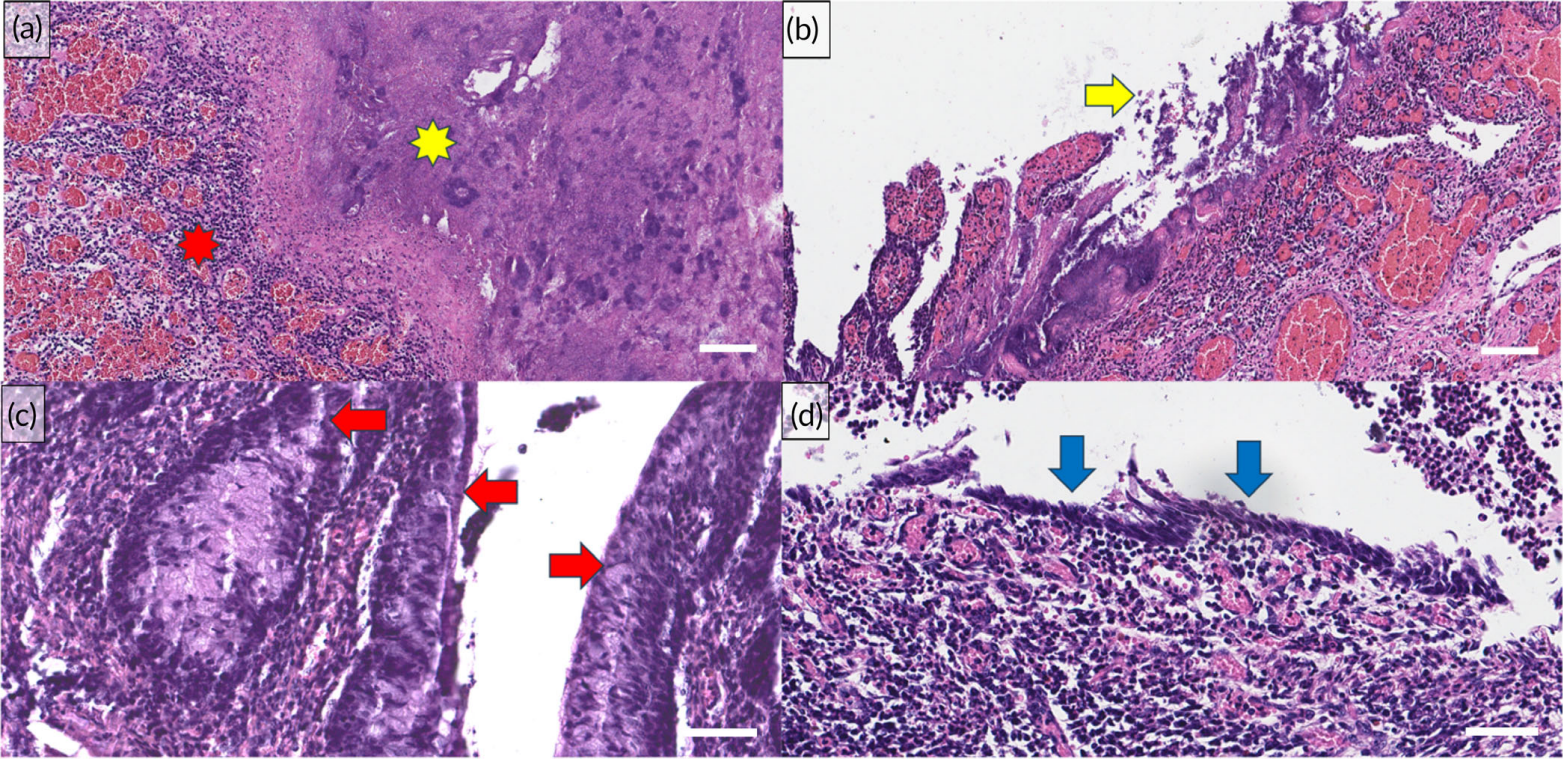
Histological evaluation of a cross‐sectional tracheal segment 2 months after application of the prosthesis: hematoxylin and eosin stains. (a) Below the prosthesis: debris and necrotic material (yellow asterisk) are stratified over a layer of inflammatory infiltrate (red asterisk), constituted prevalently by neutrophils, with focal lymphoid aggregates and occasional multinucleated giant cells, consistent with a chronic foreign‐body‐type reaction. (b) Adjacent to the prosthesis: erosions and necrotic changes of the epithelium are visible (yellow arrow), at higher magnification. (c) Mucinous metaplastic changes of the adjacent epithelium (red arrows), next to the erosions. (d) Below the prosthesis, few foci of epithelial regeneration (blue arrows). Scale bar 100 μm (a, b), 50 μm (c, d).

Histological analysis revealed extensive replacement of the defect area by exuberant granulation tissue, composed of proliferating fibroblasts and actin‐positive myofibroblasts, admixed with abundant neovascularization and edematous stroma. The inflammatory infiltrate was dense and polymorphous, with a predominance of neutrophils but also numerous lymphocytes and macrophages; focal lymphoid aggregates and occasional multinucleated giant cells were observed, consistent with a chronic foreign‐body–type reaction (Figure 7a).

The epithelial lining was largely absent, showing extensive erosion and necrosis with overlying fibrin and neutrophilic microabscesses (Figure 7b). Residual epithelium exhibited frequent mucinous metaplasia (Figure 7c), while regenerative attempts were sporadic and incomplete, limited to focal basal cell hyperplasia and small clusters of immature ciliated respiratory epithelium. Importantly, no specimen achieved continuous epithelial coverage (Figure 7d).

These qualitative findings were supported by semi‐quantitative scoring, which confirmed marked granulation tissue formation (mean 2.5 ± 0.6) and minimal epithelial regeneration (mean 0.25 ± 0.5) (Table S1, Supporting Information).

## 3 DISCUSSION

This pilot study was performed to determine the feasibility of partial airway replacement with a novel composite prosthesis. The inner layer was made of a non‐resorbable material designed to provide optimal mechanical properties. The external layers were made of a resorbable material, which has previously been shown to be biocompatible when grafted inside the airway.^13^ Overall, the results of this study suggest that modifications to either the prosthetic material or the surgical approach are needed before further experiments can be conducted. In fact, while the majority of animals survived to the predetermined endpoint of 60 days, stenosis, granulation tissue formation, and prosthesis displacement were observed in all cases.

Tracheal replacement represents an area of unmet clinical need. To address this, various experimental procedures are currently under investigation. Several research groups have independently performed tracheal allotransplantation; however, the procedure is not yet standardized, follow up is limited in most reports, and there is no certainty regarding the possibility of withdrawing immunosuppression at any point during the postoperative period.^17^ Martinod et al. recently published the results of airway replacement using stented aortic matrices in a cohort of 35 patients, with a median follow‐up period of almost 30 months.^4^ The authors reported excellent outcomes in terms of morbidity and mortality. However, only 10 out of 35 patients (28.6%) achieved a stent‐free survival.

The present study stems from our clinical observation that, in certain specific clinical circumstances, it is possible to replace a limited airway defect with a resorbable prosthesis.^13^ Because commercially available prostheses lack the necessary mechanical properties to replace the cartilaginous trachea, we attempted to design a novel prosthesis, with an inner, non‐resorbable layer resembling the structure of cartilaginous tracheal rings (Figure 1a). Manufacturing a completely synthetic product, we could overcome problems related to tissue availability and processing time, thereby representing an ideal off‐the‐shelf substitute for the airway.

There are only a few reports in the literature on the use of synthetic prostheses for airway repair in large animals. In 2007, Shultz et al. reported the use of a porous titanium prosthesis to replace 5 cm‐long circumferential tracheal defects in the sheep.^18^ The majority of the animals experienced fatal postoperative complications (e.g., tracheal obstruction, tracheal necrosis, pneumopathy), leading the authors to conclude that a change in the surgical protocol was necessary. More recently, Bhora et al. developed a prosthesis consisting of an external 3D‐printed PCL scaffold and an internal extracellular matrix dermal collagen layer.^19^ These constructs were used to repair 2 cm‐long circumferential defects in the cervical trachea of five Yucatan miniature pigs. Endoscopic examinations revealed significant paraanostomic granulation tissue, and overall animal survival ranged from 17 to 34 days.

Since we had no preliminary data on using this prosthesis for airway replacement, we designed a pilot study on a large animal model involving the creation of a small, window‐like tracheal defect. Experiments on small animal models (rats or mice) in the field of tracheal replacement do not provide reliable results for clinical translation.^20^ Moreover, previous airway wound healing studies have shown that the loss of airway lumen resulting from secondary healing of full‐thickness mucosal defects of the trachea is much greater for circumferential defects than for anterior patch‐like ones.^21^ Therefore, minimizing potential harm and the risk of sudden death from airway obstruction enabled us to carefully observe the process of wound healing over time using seriated bronchoscopic examinations, providing us with the maximum amount of information for further studies on longer and/or circumferential airway defects.

Furthermore, it should be observed that this anterior tracheal defect model has an intrinsic clinical relevance due to its similarity with a post‐tracheostomy defect. Currently, many patients who have a long‐term tracheostomy cannot be decannulated^22^ or suffer from complications after decannulation such as persistent tracheocutaneous fistula or secondary tracheal stenosis.^23^, ^24^ In such cases, the possibility of patching the anterior airway with an off‐the‐shelf tracheal substitute may represent an ideal therapeutic option.

Overall, despite achieving animal survival to the predetermined endpoint in most cases, we believe that airway healing was unsatisfactory for a combination of reasons. First, the cartilaginous defect underwent progressive contraction over time, a process that could not be prevented by the prosthesis. This healing process led to loss of airway lumen and distortion of the prosthesis itself. Second, the resorbable layer, which was expected to gradually absorb and provide a scaffold for epithelial regeneration, had completely dissolved by the 15th postoperative day. This exposed the inner layer of non‐resorbable material before any kind of tissue ingrowth across the mesh could occur. In fact, despite the open mesh design of the prosthesis and an adequate blood supply provided by the pedicled flap, epithelial regeneration was only inconsistently observed (mean 0.25 ± 0.5 on a 0–3 scale). We believe that the early degradation of the resorbable layers of the flap caused the loss of the scaffold, which was necessary to support cell repopulation and re‐epithelization. Third, the formation of granulation tissue along the edges of the defect and the intense inflammatory infiltration, which was detected histologically, hints towards a poor integration of the synthetic prosthesis with host tissues. This poor integration probably also explains why the prosthesis, which initially was sutured on the outside of the airway, was progressively dragged inside of the lumen in all cases, a phenomenon that was interpreted as a foreign body‐type inflammatory response.

Although we did not investigate the underlying molecular mechanisms, the histologic findings enable consideration of several broad biological processes that might have contributed to this evolution. The coexistence of acute and chronic inflammatory components may be indicative of a persistent injurious stimulus, whereas the stromal changes may represent nonspecific features of ongoing tissue remodeling or repair. Similarly, the incomplete epithelial regeneration may point to a limited ability of the mucosa to re‐establish a continuous epithelial lining.

A schematic representation of the mechanisms here just described, and their temporal evolution is reported in Figure 5d. Although still unsatisfactory, we consider the obtained results of considerable importance for a pilot study, highlighting the importance of the synthetic supporting structure in tracheal replacement. The majority of the treated animals did not show any respiratory distress throughout the experiment and no complications linked to mechanical failure of the prosthesis were reported. Indeed, the designed architecture composed of alternating dense sections and open, flexible meshes has proven successful in maintaining the mechanical integrity of the airway required for animal survival. The mechanical properties of the rigid polymeric rings, together with the non‐degradability of the elastomeric constituent materials, guaranteed the supporting structure's long‐lasting and efficient mechanical stability required for our pilot study. It is worth mentioning, however, that further iterations reducing the mismatch between native tissue and synthetic components should be performed to achieve the perfect balancing between supporting performance and mechanically mediated local inflammation. The mechanically mediated component of inflammation could not be investigated independently by other factors, such as the geometry, and should be investigated more thoroughly in future studies stemming from the presented results.

Unquestionably, the biological performance of the prosthesis is the major shortcoming of our approach. In this regard, although we choose medical grade PEBAX, Gore Bio‐A, and PCL, three polymers known or certified as biocompatible to minimize any adverse events, we observed subpar performance also confirmed during histological examinations. Further studies are still needed to completely dissect the complex interaction between the native tissue and the synthetic implant, which is not limited to the material compatibility. The mechanical interaction, the degradation kinetic of the external polymer, and the fixation strategy, as well as the impaired barrier function of the epithelium‐lacking defect, all deserve to be comprehended at a deeper level to assure the clinical application. Nonetheless, recognizing the main players and the shortfall in the application of an off‐the‐shelf tracheal partial implant in a clinical‐relevant setting properly represents the objective of a pilot study. Indeed, we believe that our investigation, characterized by a large animal setting and lacking any exogenous cell or complex and long heterotopic transplantation, as often proposed in literature, offers valuable preliminary data to target the translational readiness of the approach.

Based on the findings of this study, we believe that there are several ways to improve the outcomes of prosthetic airway repair. Wound contraction is an inevitable phenomenon in non‐epithelialized defects.^21^ Therefore, grafting epithelial tissue on the inner surface of the prosthesis has been proposed as a strategy to prevent complications such as shrinkage of the tracheal lumen or prosthetic infection.^18^ However, the survival of the graft depends on an adequate blood supply, which may be absent during the first weeks after implantation. An alternative method to ensure a patent lumen would be to temporarily stent the airway, as suggested by the clinical experience of Martinod et al.,^4^ but the ideal stent type and the duration of stenting remain to be determined.

**FIGURE 8:**
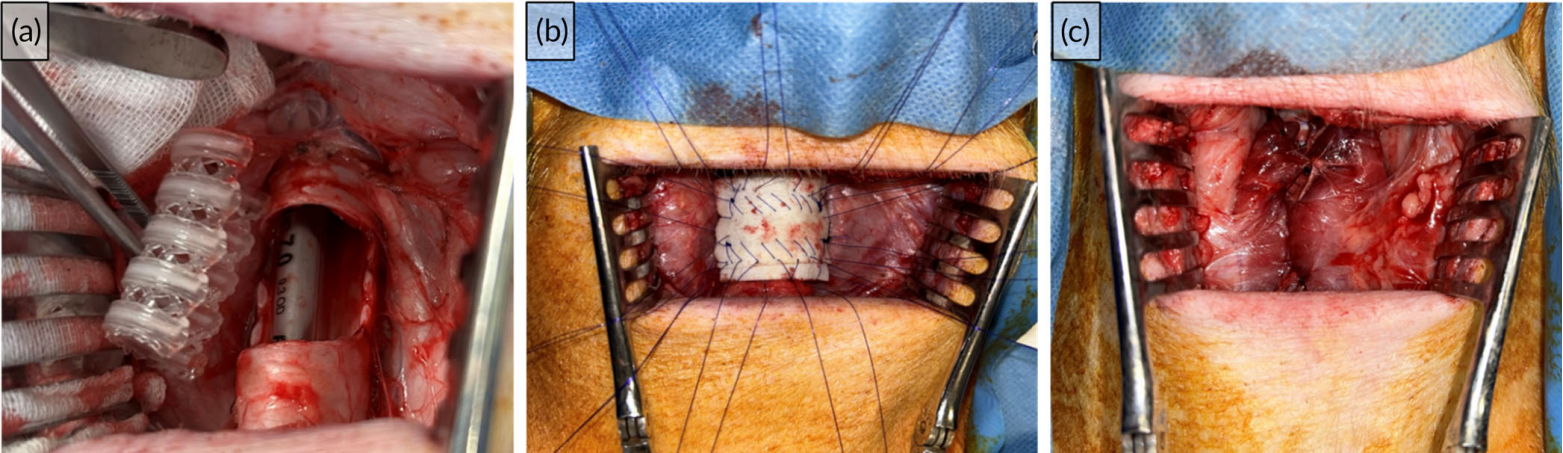
Prosthesis application on surgically induced tracheal defects. (a) A 3 cm window defect is created on the anterior side of the trachea, and the prosthesis size is chosen accordingly. (b) The composite prosthesis is fixed suturing the edges to the trachea with interrupted polydioxanone sutures. (c) The composite structure is overlaid with a pedicled flap of strap muscles.

Displacement and distortion of the prosthesis may also have been due to an inadequate anchoring strategy despite the meticulous surgical technique of fixation (Figure 8). Improvement in this aspect may be achieved by increasing the overlap area between the prosthesis and the healthy tracheal tissue, so as to make any sliding inside the edges of the window defect unlikely.

We believe that the prosthesis presented here could achieve appropriate regeneration and improved performance by experimenting with different materials for both the external layers and the internal structure. Specifically, slower degradation of the external layer could benefit the dynamics of epithelium regeneration providing a stable support throughout the healing process. Moreover, enhancing tissue ingrowth inside the prosthesis could be achieved by a further optimization of the structure porosity and integrating a controlled release of biomolecules that could induce re‐epithelization, supporting the healing process over a prolonged period, such as basic fibroblast growth factor (bFGF), epidermal growth factor (EGF), or insulin‐like growth factor type‐1 (IGF1).^25^, ^26^, ^27^, ^28^ Finally, combining synthetic materials with natural polymers, such as hyaluronic acid and collagen or bacterial cellulose, which are widely used in clinical applications for epithelial regeneration,^29^, ^30^, ^31^, ^32^ could further improve the interaction between native tissue and the prosthesis. This would reduce the stimulation of the immune system while maintaining a humid environment.

In conclusion, this study produced only partially satisfactory results regarding the use of a synthetic prosthesis for anterior trachea defect repair in a large animal model. While the mechanical properties of the PEBAX mesh successfully maintained a patent airway, early resorption of the external layer, wound contraction, granulation tissue formation, and distortion of the non‐resorbable layer resulted in variable degrees of stenosis in all cases. Further studies are necessary before the ambitious goal of creating an ideal “off‐the‐shelf” prosthesis for airway replacement can be achieved.

## 4 MATERIALS AND METHODS

### 4.1 Fabrication of the prosthesis

The semi‐rigid inner structure was designed using SEL Program Generation Software (IAI America), from which the print coordinates were obtained, and the output file was subsequently post‐processed to produce the final Gcode using a custom Python script. 3D printing via FDM was performed using an AXO A3 printer (Axolotl Biosystems). PEBAX 2533 MED (Arkema) and Purasorb PC17 (Corbion) in pellet form were loaded into separate printheads and heated to 160 and 135°C, respectively, to allow extrusion. A nozzle diameter of 0.1 mm was used for both materials. Printing speeds were set at 4 and 2 mm/s for PEBAX and PCL, respectively.

The multimaterial construct was fabricated on the planar printing bed of the 3D printer and curved subsequently by wrapping it around a stainless‐steel cylinder and heating it at 50°C for 20 min.

The final prosthesis shape was that of a semi‐cylinder, curved to an angle of 210°, with a length of 4 cm and an inner diameter of 1.8 or 2.1 cm.

The day before surgery, the constructs were sterilized by exposure to UV light for 30 min and then stored in phosphate‐buffered saline solution with 1% penicillin/streptomycin until surgery.

At the time of surgery, the most suitable size was selected based on the animal's tracheal diameter; then, the inner layer was covered both in its internal and external surfaces by a sheet of Gore Bio‐A tissue reinforcement. The three layers were fixed together by two absorbable running 3‐0 polydioxanone sutures (PDS; Ethicon, Somerville, NJ).

### 4.2 Tensile testing of the native tracheal tissue and the prosthesis

Tensile tests on both native tracheal tissue and the composite prosthesis were performed to characterize and compare their mechanical behavior.

Tracheal samples were obtained from three cartilaginous half‐rings extracted from anterior sections of porcine tracheal tissue from four animals; a gauge length of 6 mm was selected for all samples to ensure a mean aspect ratio (length/width) of about 3. The effective width and thickness of the samples were measured through ImageJ from top and side photos of the specimens.

Prosthesis samples simulating sections of cartilaginous rings were extracted from a single composite graft and consisted of individual rigid segments of PCL and PEBAX.

For the mechanical tests, Bose ElectroForce® Planar Biaxial Test Bench instrument (TA Instruments, New Castle, DE) was used under displacement control.

For tracheal samples, the loading protocol consisted of five consecutive loading–unloading cycles, each reaching a maximum nominal strain of 10% at a constant strain rate of 1% s^−1^. From each test, the tensile curve relating to the fifth cycle was considered for further elaborations, as representative of the mechanical response of the tissue under stable conditions. Prosthesis samples were tested through a single tensile test up to a maximal nominal strain of 10% at a constant strain rate of 1% s^−1^.

Nominal strain (*ε*) was calculated as the ratio between the grip displacement and the sample's initial gauge length, whereas nominal stress (*σ*) was obtained by dividing the recorded force by the sample's initial transversal area. From tensile curves of each specimen, the secant modulus *E*_*s*_ was calculated as the slope of the straight line drawn from the origin of the stress–strain diagram and intersecting experimental data at 10% strain.

### 4.3 Animal procedure

The study protocol was approved by the local animal welfare body and the Italian Ministry of Health (387/2021‐PR), and all procedures were performed in accordance with local regulations on animal welfare and with ARRIVE guidelines (Figure S1). Because it is a pilot study, no formal sample size estimation was done, in compliance with this kind of study design, which is meant to inform about the feasibility of a specific novel treatment on a small sample size.^33^ This experiment aimed to investigate the effect of the interposition of a novel prosthesis on an anterior, window‐like tracheal defect. Because in clinical practice there is currently no accepted standard procedure or material that can be used for this purpose, no control group was used.

Four 5‐month‐old female domestic pigs (*Sus scrofa domesticus*) weighing 55 ± 4 kg were used as recipients for testing this novel prosthesis. We chose female pigs in order to reduce any heterogeneity in the animals' growth rate or other potential sex‐related confounders. Animals, preliminarily housed in multiple pens at the porcine experimental facility of the Department of Veterinary Medical Sciences of Bologna University, were fed twice daily with a standard commercial diet and kept at a 12/12 light/dark cycle.

The day of surgery, animals underwent general anesthesia after 12 h fasting. Pigs were administered intramuscularly (IM) with a combination of tiletamine‐zolazepam (3 mg/kg) and dexmedetomidine (15 μg/kg) and, upon achievement of lateral recumbency, general anesthesia was induced intravenously (IV) with propofol (2–4 mg/kg, titrated to effect). After orotracheal intubation, anesthesia was maintained using sevoflurane (2–4%) in a 1:1 mixture of oxygen and medical air. An extra‐long orotracheal tube was fashioned by connecting two tubes with sterile surgical tape so as to have the cuff lying as distal in the trachea as possible. Animals were mechanically ventilated in VCV mode (volume‐controlled ventilation): tidal volume was set at 6–7 mg/kg, respiratory rate was adjusted to normocapnia. Systemic analgesia was granted by constant rate infusion (CRI) of fentanyl (6–10 μg/kg/h). The animals were placed in supine position; the neck was scrubbed with alcohol and povidone iodine and draped in a sterile fashion. Following collar cervicectomy, the trachea was exposed from cricoid to sternal notch. Then, an incision was made below the second tracheal ring, and a window defect of approximately 3 cm and half the tracheal circumference was created (Figure 8a). The prosthesis was sized and fashioned as described above; then, it was trimmed to be slightly larger than the defect and fixed to tracheal edges by interrupted 3‐0 PDS sutures (Figure 8b). Airtightness was tested by filling the operative field with saline and deflating the cuff of the endotracheal tube. Then, the prosthesis was buttressed by apposition of a pedicled flap of strap muscles (Figure 8c), and the wound was closed in standard fashion. At the end of the procedure, the animals were placed in prone position, extubated, and a bronchoscopy was performed to check the initial status of the airway. For the latter, anesthesia was maintained using a propofol CRI (0.1–0.2 mg/kg/min). Bronchoscopy was performed using a flexible 6 mm Pentax EG‐1870K endoscope with EPK‐i5000 videoprocessor. Pigs were then recovered in a monitored setting and subsequently moved to individual pens in the animal facility. Perioperative antibiotics (enrofloxacin, 2.5 mg/kg IM SID) and pain medications (flunixin‐meglumine, 2.2 mg/kg IM SID) were managed and administered by the veterinary team for a minimum of 5 days.

### 4.4 Postoperative monitoring

Monitoring was performed daily. Data on overall status, weight, and respiratory status were collected. Follow‐up bronchoscopies were performed using the same equipment as previously described, under deep sedation, at 15, 30, and 60 days from surgery. During the examination, the healing of the defect, the status of the prosthesis, and the size of remaining tracheal lumen were assessed. The grade of stenosis observed at postoperative bronchoscopies was expressed as the percentage of patent lumen at the level of the prosthetic repair divided by the lumen of the normal trachea below the repair. Similarly, resorption of the external layer was expressed as the percentage of exposed prosthesis relative to the total surface of the patched area. On the same days, blood was collected for complete blood count (K3 EDTA tubes) and clinical chemistry (cloth activator tubes). Calculated white blood cells are expressed as absolute value of cells/mm^3^ blood and (%) of baseline value. An aliquot of plasma, obtained upon centrifugation of a K3 EDTA tube, was stored at −80°C for IL‐6 quantification. The latter was performed, according to the manufacturer's instructions, using a commercial ELISA kit (Porcine IL‐6, cod. ESIL6, Invitrogen). Euthanasia was performed at 60 days by means of barbiturates overdose (sodium thiopental, 40 mg/kg).

### 4.5 Postmortem examinations

Soon after euthanasia, the tracheal segment was excised with the overlying muscle tissue, immersed in formalin solution, and carried to the pathology laboratory.

Before histological examination, the tracheal specimen underwent comprehensive macroscopic assessment. The longitudinal and transverse diameters of the tracheal segment were precisely measured, and photographs were taken to document the inner and outer surfaces. The specimen was inspected for structural alterations, including tissue deformation, erosion, necrosis, or discoloration, and all abnormalities were systematically recorded. The position and integration of the prosthesis were carefully evaluated, with any signs of dislocation, detachment, or lack of tissue integration noted. Following macroscopic evaluation, the specimen was prepared for histological analysis. Longitudinal views of the inner tracheal surface were prepared by making an incision along the posterior aspect of the trachea, allowing the specimen to be laid flat for detailed visualization. Transverse views were obtained by sectioning the trachea perpendicularly to its longitudinal axis at regular intervals. For each transverse section, three representative fragments were systematically sampled from distinct zones to ensure comprehensive coverage of the tracheal architecture. These fragments were fixed in formalin, embedded in paraffin, and processed for routine histopathological examination using hematoxylin and eosin (H&E) staining. Semi‐quantitative histological assessment was also performed to support the descriptive analysis. Granulation tissue and epithelial regeneration were independently graded by two blinded observers using a 0–3 scale (0 = absent, 1 = mild, 2 = moderate, 3 = marked) based on the extent and cellularity observed in H&E‐stained sections. Mean values were calculated across all animals.

### 4.6 Statistical analysis

Due to the small sample size, only descriptive statistics were reported. All numerical data are summarized as mean ± SD. For the mechanical characterization of the native porcine trachea, three samples were tested from four different animals while for the curved prosthesis, three samples were obtained and tested. Data for both the native tissue and the prosthesis are reported as the mean values and the standard deviations in MPa calculated from the performed mechanical tests. The grade of stenosis, reported as the percentage of patent lumen, as well as the resorption of the external layer and the white blood cells are represented as the mean value (±SD) calculated from the four treated animals at the examined timepoints.

## AUTHOR CONTRIBUTIONS

**Marco Mammana**: Conceptualization; investigation; writing – original draft; writing – review & editing; project administration; methodology. **Alessandro Gandin**: Investigation; writing – original draft; writing – review & editing; funding acquisition; project administration. **Giovanni Zambello**: Formal analysis; data curation; investigation. **Margherita Pelosin**: Investigation; software; writing – original draft. **Alberto Elmi**: Investigation; resources; conceptualization. **Domenico Ventrella**: Investigation; resources; methodology. **Silvia Todros**: Methodology. **Veronica Torresan**: Methodology; investigation; writing – review & editing. **Federica Pezzuto**: Formal analysis; investigation; writing – original draft. **Marco Pietra**: Investigation; conceptualization; formal analysis. **Noemi Romagnoli**: Investigation; formal analysis. **Andrea Dell'Amore**: Supervision; visualization. **Maria Laura Bacci**: Supervision; resources. **Fiorella Calabrese**: Supervision; methodology. **Giovanna Brusatin**: Funding acquisition; supervision; project administration; writing – review & editing; resources. **Federico Rea**: Funding acquisition; validation; supervision; conceptualization; data curation.

## CONFLICT OF INTEREST STATEMENT

The authors declare no conflicts of interest.

## Supporting information


**Data S1.** Supporting Information.

## Data Availability

The data that support the findings of this study are available from the corresponding author upon reasonable request.
